# An introductory review of parallel independent component analysis (p-ICA) and a guide to applying p-ICA to genetic data and imaging phenotypes to identify disease-associated biological pathways and systems in common complex disorders

**DOI:** 10.3389/fgene.2015.00276

**Published:** 2015-09-07

**Authors:** Godfrey D. Pearlson, Jingyu Liu, Vince D. Calhoun

**Affiliations:** ^1^The Olin Neuropsychiatry Research Center, Institute of Living, HartfordCT, USA; ^2^Department of Neurobiology, Yale School of Medicine, Yale University, New HavenCT, USA; ^3^Department of Psychiatry, Yale School of Medicine, Yale University, New HavenCT, USA; ^4^Department of Electrical and Computer Engineering, and The Mind Research Network, The University of New Mexico, AlbuquerqueNM, USA

**Keywords:** multivariate, imaging genetics, parallel independent component analysis, common disease, common variant, genetic risk

## Abstract

Complex inherited phenotypes, including those for many common medical and psychiatric diseases, are most likely underpinned by multiple genes contributing to interlocking molecular biological processes, along with environmental factors (Owen et al., 2010). Despite this, genotyping strategies for complex, inherited, disease-related phenotypes mostly employ univariate analyses, e.g., genome wide association. Such procedures most often identify isolated risk-related SNPs or loci, not the underlying biological pathways necessary to help guide the development of novel treatment approaches. This article focuses on the multivariate analysis strategy of parallel (i.e., simultaneous combination of SNP and neuroimage information) independent component analysis (p-ICA), which typically yields large clusters of functionally related SNPs statistically correlated with phenotype components, whose overall molecular biologic relevance is inferred subsequently using annotation software suites. Because this is a novel approach, whose details are relatively new to the field we summarize its underlying principles and address conceptual questions regarding interpretation of resulting data and provide practical illustrations of the method.

## Introduction

“…**essentially all models are wrong, but some are useful**”

George Box and Norman Draper in ‘Response Surface Methodology’

(Box and Draper, 1987)

Just as we would not argue that complex traits at a brain level were represented by a single region or cell type, but rather by an interplay between multiple networks (Calhoun et al., 2009a; Williamson and Allman, 2012), similarly complex inherited phenotypes such as many common medical and psychiatric diseases are unlikely to be underpinned by a single gene, but rather by multiple genes contributing to interlocking molecular biological processes in association with environmental factors (Sullivan, 2012; Ridge et al., 2013). However, despite general agreement on this context, current strategies used to genotype complex, inherited disease-related phenotypes are almost exclusively predicated on univariate analyses. Such approaches include genome wide association studies (GWAS) that most often identify single risk-related SNPs or loci, rather than the underlying biological pathways (Meda et al., 2014). While GWAS of very large samples have usefully detected associations of common SNPs and of common neuropsychiatric disorders, they are much less useful in revealing those pathophysiological molecular mechanisms necessary to guide development of novel treatment approaches. Strategies employed in an attempt to move the field beyond this logjam include at the phenotype level, classifiers that cross diagnostic boundaries (such as psychosis) or putatively simpler markers of biological disease predisposition such as endophenotypes (Tamminga et al., 2014). At the genotype level, this article explores the use of multivariate analysis strategies, in particular parallel independent component analysis (i.e., simultaneous combination of SNP and neuroimaging information). The output of these analyses typically yield clusters of functionally related SNPs that are statistically correlated with phenotype components and whose overall molecular biologic relevance can be inferred through using annotation software suites such as BioCarta^1^ or KEGG^2^. We believe that multivariate approaches like parallel ICA (p-ICA) are promising, but they are not yet familiar to many investigators. Thus this review attempts to summarize their underlying principles and use, to address conceptual questions that arise regarding their interpretation and to provide practical illustrations.

In order to achieve this, we first recapitulate briefly arguments regarding the genetic architecture of common complex medical disorders, difficulties encountered in applying univariate models to these illnesses and their appropriateness as targets of study for multivariate genetic strategies. To expand the latter point, we review the benefits of network-based approaches to study complex inter-related patterns, and argue that in the case of multi-model imaging and genetics data, it is significantly more informative to analyze these domains jointly rather than separately. We next provide a series of sections detailing an overview of p-ICA approaches, with examples and numerous, detailed practical instances. Finally, we address the issue of replication when employing these approaches, as well as ongoing issues in need of solution and summarize some future directions for p-ICA.

## Common Disease Common Variant (CDCV) Models and Their Validity

To date, p-ICA approaches have been applied most often to neuropsychiatric disorders, although as we explain below, common complex medical disorders in fact cover many disease domains. As a general rule, major psychiatric disorders including schizophrenia and autism display similar inheritance patterns to common medical conditions, e.g., type-II diabetes, asthma, or inflammatory bowel disease, characterized by both fairly high heritability and genetic complexity (Pearlson and Folley, 2008). This view is somewhat oversimplified, as more exact heritability measurements can be given, see, for example, the paper from (Lee et al., 2013). Despite high heritability, generally, most affected individuals have negative family histories of the disorder, and simple Mendelian genetic models are inapplicable (Risch, 1990). Remaining genetic inheritance models subsume various possible combinations of number, frequency, penetrance, and effect size of genetic risk alleles (Wray and Visscher, 2010), including numerous common genetic loci.

In these conditions, cumulative evidence suggests that a common disease common variant model (CDCV) still accounts for many cases of these disorders. This model presumes multiple, [likely hundreds or thousands (Wray and Visscher, 2010)] of possible variants of low individual risk, that both evade the threshold of detection and have sufficiently weak individual effects to escape elimination by natural selection. Another statement of this hypothesis is that some among the common genetic variants in coding and regulatory portions of genes, that are individually evolutionarily neutral or of low penetrance, in combination (either additively or multiplicatively) lead to susceptibility to complex polygenic disorders.

In addition, uncommon, non-SNP structural variants of moderate effect size [mutations/duplications/deletions, including copy number variations (CNVs)] account for a proportion of cases of schizophrenia, autism, epilepsy, and intellectual disability (O’Donovan et al., 2009), albeit rather non-specifically. Rare-variant/large effect size and CDCV models are not mutually exclusive; likely, both possess independent explanatory power and can be combined in a “mixed economy,” e.g., (Reich and Lander, 2001; Schork et al., 2009; Xu et al., 2009; Owen et al., 2010; Visscher et al., 2012). In future, it is likely that p-ICA will be applied to additional non brain-based common complex diseases.

## Univariate Models and Their Limitations

One reason that multivariate techniques such as p-ICA are gaining traction as alternate analytic techniques is due to limitations in univariate genetic models when applied to common complex disorders. Univariate approaches generally presume large subject populations (typically for psychiatric disorders in the tens of thousands) and comprehensive SNP sampling, representing a significant proportion of common genomic variation. If a major assumption of CDCV models is correct, i.e., that genes individually conveying modest risk for a disorder combine, (perhaps epistatically), then univariate GWAS-like approaches likely detect only the “tip of the iceberg,” i.e., the very small number of individual genetic loci conveying the greatest detectable disease risk in that analysis. Multiple associated genomic markers reported for particular phenotypes to date that both transcend appropriate significance thresholds and replicate in independent samples, generally explain only a small proportion of the total genetic variability, are frequently scattered across multiple genomic regions and have unclear biologic functions. The remainder of the “iceberg” remains submerged, [with the gap between phenotypic variation explained by all associated variants and estimated total heritability, termed “missing heritability” (Visscher et al., 2012)].

Because individual genes frequently participate in multiple molecular biologic pathways and often contribute to risk for several brain-based disorders, it is difficult to infer relevant functional pathway(s) from the small number of genes typically implicated in GWAS. Univariate approaches are unlikely to detect epistasis (Frazer et al., 2009); none have done so to date (Frazer et al., 2009; Ke, 2012). For both height (Visscher et al., 2012) and for schizophrenia (Purcell et al., 2009; Ripke et al., 2011) evidence points to many variants with very small effect sizes failing to reach statistical significance, even with very large sample sizes, but where significant variation is accounted for by all associated SNPs, e.g., 23% of variation in schizophrenia liability is captured by SNPs (Lee et al., 2012), mostly common causal variants, (although SNPs identified to date explain a much smaller proportion of risk). Additionally, gene–gene interactions are likely important, even for some Mendelian disorders such as Marfan syndrome (Kaiser, 2012). Investigators have used strategies including generalized additive models for GWAS (Jia et al., 2012), for gene set enrichment analysis of GWAS data, adjusting for gene length or SNP number bias, in order to help determine the underlying biological significance of multiple SNPs derived from GWAS. Such models do not address interactions among the selected SNPs (testing the lump sum significance), however. Currently, neither individual genes identified in univariate analyses nor the discovery of CNVs alone can provide details of the full “diseasome” (Frazer et al., 2009) model, (that aims to provide a comprehensive representation of the relationship between a given disease and its interrelationship with genomic factors), or provide a useful understanding of the remainder of the “iceberg,” especially so for gene–gene interactions. These deficiencies prompt a need for multivariate-based approaches, that we describe below and amongst other features are sufficiently statistically robust to be informative in smaller sample sizes [typically in the hundreds or low thousands, that fit real-world circumstances (Ripke et al., 2011; Visscher et al., 2012)].

## Benefits of a Network based Approach (Networks versus Points)

**FIGURE 1:**
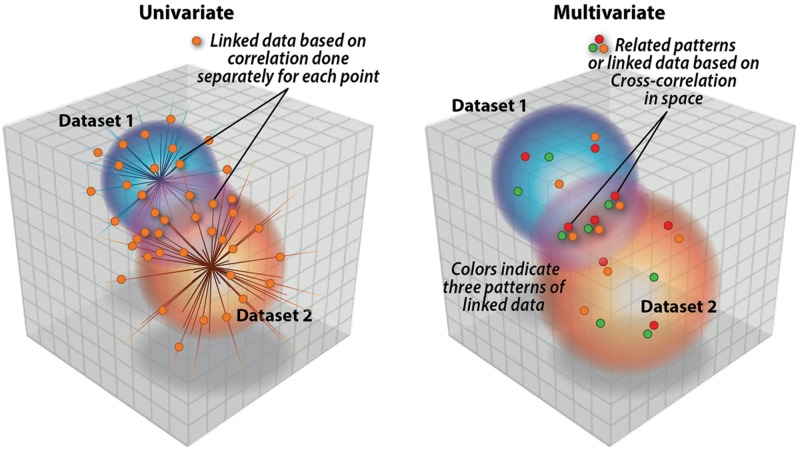
**Univariate approaches are focused on single points of relation whereas multivariate approaches like parallel ICA (p-ICA) focus on links between patterns (e.g., weighted combinations of brain regions and weighed combinations of genetic variables**.

Multivariate approaches (Calhoun et al., 2009b; Hardoon et al., 2009; Purcell et al., 2009; Vounou et al., 2010; Le Floch et al., 2012; Liu and Calhoun, 2014) have a benefit over univariate approaches as their focus is on inter-related patterns not unrelated points (see **Figure 1**). This makes them ideally suited for identifying complex, but potentially weak, effects buried in a high-dimensional data set. Another way of describing a multivariate result (or an independent component) is to call it a ‘network.’ Some explanation is needed as the term network is used widely and with varying definitions across different scientific fields. For example, in the fMRI field, network is used to designate variously (1) regions correlated with a common seed-point (Biswal, 2012), (2) temporally coherent regions from an ICA analysis (Calhoun and Adalı, 2012), or (3) a graph-based construct based on correlations among nodes and edges (which may come from voxels, regions, or components; Yu et al., 2011, 2012a,b). The first two cases provide example of a univariate approach and a multivariate approach. A simple illustrative example in (Erhardt et al., 2011) showed that in the case of a univariate approach, e.g., seed-based cross-correlation, one is only guaranteed that a given voxel is correlated with the seed, but no assurance that any of the network voxels are correlated to one another.

ICA (Comon, 1994) is a model-free/data-driven computational method, based on blind source separation, used in signal processing for separating a multi variant signal into additive subcomponents. A frequently cited example is the “cocktail party problem,” where one “voice” needs to be separated from the noisy background. ICA assumes that sub-components are statistically independent and that all but one are non-Gaussian. The non-Gaussian assumption is convenient as it also has the effect of assuming a small number of voxels and/or SNPs will have large contribution to a given component. In contrast to principal components analysis, which determines the maximal separation of components using second order statistics, ICA determines solutions that maximize independence, using higher-order statistics.

In contrast to univariate analyses, because an approach like ICA estimates all the variables jointly, by definition the voxels in the ‘network’ are functioning coherently with one another. This property of ICA methods (and in extension, p-ICA) provides three major benefits. First, it helps with interpretation, as one can accurately assume the region (or genes) in a given component covary together. Secondly, it provides robustness to noise. For example, again to draw on the fMRI example, correlation-based approaches can be ‘tricked’ by phenomena such as phase randomized noise which can appear to represent real signal (Handwerker et al., 2012). However, in the case of ICA, the assumptions are stronger in that one is identifying patterns and thus the same type of randomized noise will not resemble real signal. This is not to say that ICA-based methods are impervious to noise, but they do tend to be more robust than univariate correlation as they are working with patterns rather than just paired relationships. ICA-based methods are not the only approaches that have this advantage, for example, other multivariate approaches becoming widely used include sparse reduced rank regression (Vounou et al., 2010) and sparse canonical correlation analysis (Lin et al., 2014a,b). And finally, because the statistical testing is done at the level of networks, correction for multiple comparisons is appropriately based on the number of network tested, rather than the number of SNPs or voxels.

Such approaches are able to capture multiple links among genetic factors; this can include population effects but also weaker effects of interest or links among patterns of genetic data and patterns of phenotypic data. Finally, p-ICA enables analyses of whole-brain imaging genetics, that PLINK does not (Purcell et al., 2007). The sections immediately following lay out the theoretical background and practical implementation of p-ICA approaches.

## Why Parallel? The Benefits of Performing Data Fusion

**FIGURE 2:**
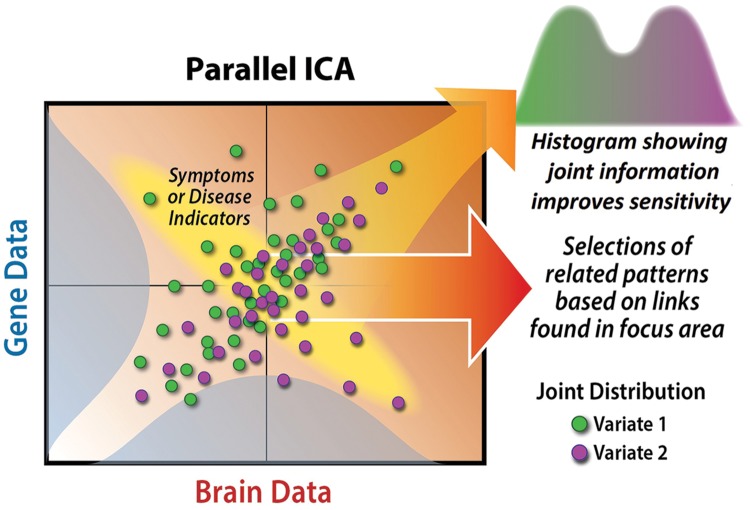
**The benefit of a joint analysis is we can capitalize on the joint distribution of (in this case) the imaging and genetic data, something that can provide a better ability to discriminate health and disease.** When we have two data sets, each with numerous variables, we could compute huge numbers of cross-correlations (adjusting for requisite multiple comparisons). Here, p-ICA displays a definite advantage, providing both a means to identify relationships among two very large data sets, while simultaneously identifying the most relevant variables representing this information, (i.e., simultaneously performing data reduction).

Why should we analyze multimodal imaging and genetics data jointly, instead of just analyzing each domain separately? A useful thought experiment is to consider identifying a single relevant genetic factor and correlating it with all brain voxels across subjects to identify a putative intermediate phenotype. This is obviously informative differently than looking separately at which brain regions show group-related activity changes and which genetic factors show group-related differences. Such an analysis is a type of data fusion, because both data sets are used to estimate a result. Such approaches can provide improved ability to distinguish patients versus controls, for example, by capitalizing on the joint information (see **Figure 2**).

## Parallel ICA vs. ICA

**FIGURE 3:**
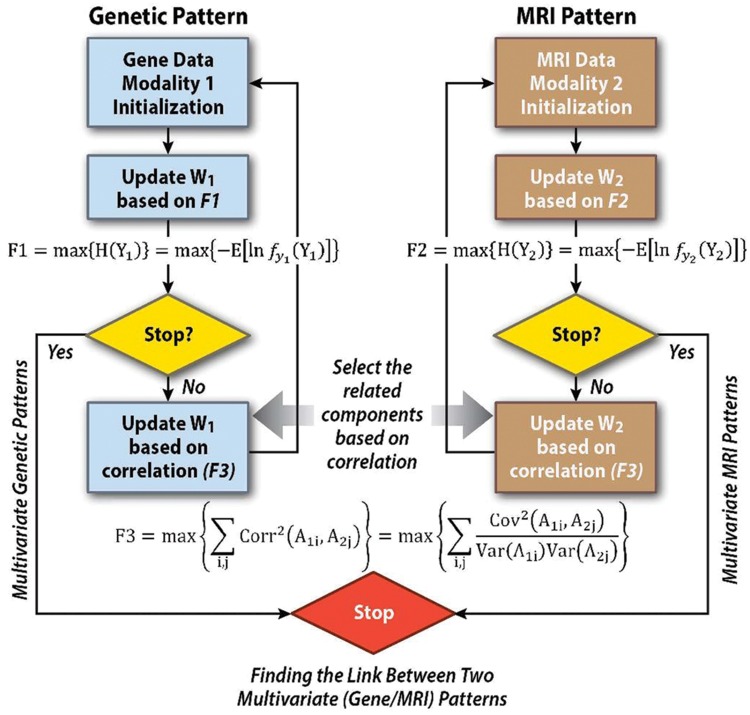
**Weighted combinations of brain regions are linked to weighted combinations of genetic variables which can then be tested for associations with variables of interest (e.g., disease status, symptoms).** Components extracted by p-ICA are a linear weighted combination of all variables. Each variable’s weight indicates its contribution to the component, and helps to interpret it. For instance, the genetic component, perhaps formed from thousands of SNP markers, is mainly contributed to by top-weighted markers. The remainder, with much lower weights do not markedly affect the component loading.

ICA is an approach that takes one data set and identifies components that are maximally independent of one another. In contrast, p-ICA extends this by applying ICA to two datasets jointly, while also incorporating a term that tries to identify links among the two datasets. In the context of p-ICA, the goal is to identify maximally independent networks from two or more data sets simultaneously, as well as identifying links among them. This is done by jointly maximizing several ‘cost functions,’ one to specify the independence among networks in each of the data sets and a second that specifies the link among networks across data sets (e.g., the correlation among pairs of networks across data sets). These steps are accomplished together in a single algorithm (see **Figure 3**: Block diagram showing the steps involved in p-ICA including (1) identification of maximally independent patterns within each modality, and (2) identification of the possible links among the multimodal patterns. The ICA optimization for genetics (mathematically described by F1, where Y1 is the genetic data) and magnetic resonance imaging (MRI; mathematically described by F2, where Y2 is the MRI data) are shown in the blue and brown boxes, respectively. W1 refers to the unmixing matrix for the genetic data and W2 refers to the unmixing matrix for the MRI data. The link among these two ICAs is described by equation F3, where Aij are the ICA loading parameters for datatype i and subject j. F1–F3 are solved for simultaneously, providing maximal independence within genetics data, MRI data, as well as the link among the two, hence the name p-ICA).

**FIGURE 4:**
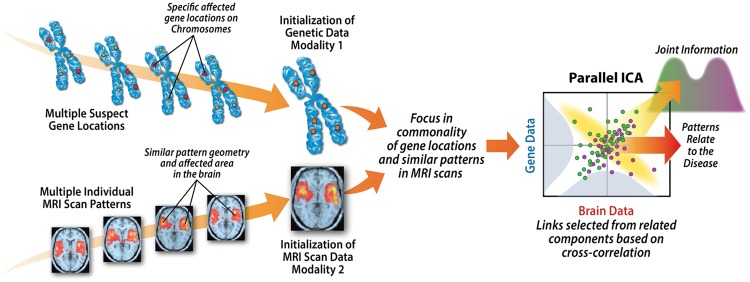
**Weighted combinations of brain regions are linked to weighted combinations of genetic variables which can then be tested for associations with variables of interest (e.g., disease, symptoms)**.

One of the reasons for a joint optimization in this way is it enable us to combine data types that have different ranges and properties, reducing it down to a maximization of two entropy terms (one for neuroimaging, one for SNPs) each of which controls the independence among the components within each data-type, and a cross-correlation among each data-type’s component loadings, providing a normalized measure of the strength of the relationship among each modality for each component pair, (see **Figure 4**).

Parallel-ICA is designed for multimodal processing, and extracts components using an entropy term based on information theory to maximize independence and enhances interconnections among components by maximizing the linkage function in a joint estimation process. This technique can identify and quantify associations between two sets of features (e.g., functional MRI, structural MRI, genes, behavior, etc.) and determine significance, typically in a patient-versus-control context embedded in the components.

Consider a specific case in which we have an fMRI contrast image (consisting of the percent signal change in each voxel associated with a given task) and a SNP array (where values can be coded as -1, 0, or 1 to reflect homozygous or heterozygous states [e.g., AA, (aA or Aa), and aa] (Calhoun and Adalı, 2009). In this case, in order to provide a more tractable model, the fMRI data are reduced to a contrast map instead of including the full space-by-time fMRI data. This is called a ‘feature-based’ analysis, where a feature of interest is extracted from the original data and then submitted to the data fusion algorithm (Calhoun and Adalı, 2009). Though it is also desirable to consider a model that can capture the full fMRI data and the SNP data at once, the feature-based approach makes the problem considerably easier and provides a relatively parsimonious solution still enables us to capture the joint information among multiple high dimensional data sets. Feature-based solutions have been shown to work well with multiple types of data (Calhoun and Adalı, 2009), and enable one to emphasize a particular, salient aspect of the data while still capturing relevant information such as the presence of major temporally coherent networks in the fMRI data (Calhoun and Allen, 2013). In general, a feature includes multiple variables (e.g., voxels or SNPs) for each subject and is organized for a group of subjects as a subject-by-feature matrix. The associations between the two types of data are made based on inferring a correlation or link across subjects (e.g., subjects with a certain linear combination of SNPs also tend to show a certain linear combination of voxels). Such an approach enables p-ICA to be used with various types of modalities including fMRI (Liu et al., 2009b), structural MRI (gray matter, white matter; Chen et al., 2013a), electroencephalography (EEG; Liu and Calhoun, 2007), gene expression, methylation (Liu et al., 2010, 2014), or metabolomics, quite straightforwardly.

## An Informative Illustration of Parallel ICA and Common Preprocessing

Let us start with a simple case where we have 10 SNPs for each of 100 subjects. The SNPs are represented by a matrix X_s,i_ where *s* indicated the SNP number (from 1 to 10) and *i* indicates the subjects (from 1 to 100). Consider also an fMRI contrast image with 10 voxels represented by a matrix Y_v,i_ where *v* represents the voxel number (from 1 to 10) and *i* represented the subjects (from 1 to 100). In the case of p-ICA we want to find a representation of the data that includes SNP components, each of which can be considered a ‘pattern’ of SNPs and fMRI components, each of which can be considered a pattern of fMRI voxels. Mathematically we can write X = AS, Y = BT where *A* and *B* are called mixing matrices that indicate the degree to which each subject’s SNP and fMRI data are represented by the respective components. The components are noted as S and T for SNPs and fMRI voxels, respectively. The link, more precisely the correlation between mixing matrices A and B, reflects whether or how strongly a SNP component is associated with a fMRI component in a way that how the SNP component is expressed in subjects is related to how the fMRI component is expressed in subjects. The p-ICA models suggest that analyses work most validly within certain ratios of genotype-to-sample size, limited by detection power for reasonable effect sizes (Liu et al., 2008). Currently more than 5 million SNPs across the whole genome can be genotyped simultaneously; sequencing can achieve about 3 billion genetic markers. However, not all available markers are useful for association analyses. Thus, several standard preprocessing steps should be applied before implementing association tests, to minimize low quality data and remove irrelevant variables, using suggested guidelines for quality control of genetic data (Anderson et al., 2010).

One recent illustrative example of an interesting, novel result yielded by p-ICA derives from analysis of a functional functional magnetic resonance imaging (MRI) data set in a genotyped population of ∼550 psychiatric probands suffering from schizophrenia or psychotic bipolar illness, plus healthy community controls who underwent resting state MRI scans to identify imaging-genetic relationships (Meda et al., 2014). It is known that the brain’s default mode network (DMN) is highly heritable and shows abnormalities in many psychiatric disorders including schizophrenia and bipolar illness. However, genes underpinning DMN patterns in healthy and ill individuals remain mostly unknown. In the above investigation, P-ICA subdivided the DMN into five sub-networks, that were significantly associated with five different SNP components. Several of the highest-ranking SNPs across these networks derived from genes that had previously been identified as contributing risk to psychosis and/or mood disorders in large-scale GWAS studies. More pertinently, global enrichment of SNPs from the genetic components highlighted processes implicating specific neurotransmitter, developmental and other relevant central nervous system biologic pathways including NMDA-related long-term potentiation, axon guidance, synaptogenesis, immune-mediated neuronal response signaling and protein kinase A. Highly enriched network processes included cellular signaling, neurodevelopmental and transport networks containing axonal guidance and cell adhesion processes, consistent with pre-existing hypotheses implicating membrane scaffolding and neuronal cell adhesion proteins as important contributors to susceptibility for both schizophrenia and bipolar illness. Thus, in addition to confirming several known schizophrenia risk genes previously derived from GWAS, the study also highlighted additional genes acting in synchrony that acted as signposts to biologic processes that are consistent with leading hypotheses in the etiology of psychotic illnesses.

## Common Practical Issues in Implementing p-ICA

After initial preprocessing and quality control steps, hundreds of thousands of SNPs can remain. Different dimension reduction strategies can be applied, each with accompanying limitations. Since adjacent SNPs are likely inherited coherently forming a linkage disequilibrium (LD) block, SNPs within such blocks present redundant information from an association viewpoint and can be reduced by including one SNP within each LD block. However, we do not know which SNP is more likely to be causal, thus, one must check the removed SNPs when interpreting findings from reduced data. Otherwise, grouping and/or selection of SNPs can be based on functional locations such as exomes or promoter regions, assuming known functional active regions are a specific focus of the study. Second, based on hypotheses and known gene/pathway functions we can limit analyses to SNPs within specified pathways or genes, e.g., KEGG pathways/annotations, or restrict SNPs to those deriving from genes with known annotations/functions. Then, association analyses become partially hypothesis-driven and partially data-driven, (located along the spectrum between completely data-blind ICA-based models versus more-informed hypothesis-driven approaches). Limiting SNP input to biological pathways does not exclude finding new disease-related genes, but lowers the odds of discovering a true positive located in a previously unknown biological pathway. The penultimate approach selects data available from large, publicly available genetic consortia, such as the Psychiatric Genomics Consortium (PGC)^3^, limited to SNPs with potential impact on phenotypes of interest. For instance, after conducting univariate tests on individual SNPs associated with particular diagnoses in a large population sample, we select SNPs with relatively liberal association levels with diagnoses (e.g., *p* < 0.05 uncorrected) and perform multivariate analyses with another intermediate phenotype like brain imaging, in a different, smaller sample. Though still pre-filtering, this involves relatively less bias and less weight on prior predictions. Finally, p-ICA restricted to genes previously discovered or linked to particular diseases (e.g., the Broad Institute’s Psych Chip^4^), could discover new multivariate relationships between genes and traits without facilitating new gene discovery. The Psych Chip is an inexpensive genotyping chip manufactured by Illumina covering ∼240 K tag SNP markers and equal number of exome-focused markers, in order to augment coverage of common SNPs in psychiatrically relevant regions while simultaneously augmenting coverage across rare CNVs to focus on psychiatrically relevant very rare variants not present on the Exome chip. It contains ∼50 K common variants (GWAS) relevant to multiple common psychiatric disorders, based on information from the Psychiatric Genetics Consortium (PGC^5^), whose purpose is to conduct mega-analyses of genome-wide genetic data for psychiatric disorders.

The next sections reviews series of other important steps in conducting a successful p-ICA.

### Selecting the Optimal Number of Components

In practice, the number of components embedded in genetic or phenotypic data is usually estimated beforehand, and associations between components then evaluated through p-ICA. Different strategies are applied to optimal estimation of component number within any observations. The information theory-based Akaike information criterion (AIC), model -selection approach (Akaike, 1974) is used to improve estimation of the number of independent components. Similarly, minimum description length (MDL) measures constitute another information theoretic approach for model selection which determines the model order by compressing the data-based on regularities while avoiding overfitting. MDL approaches are designed to strike a balance between variance explained by components and degree of freedom imposed by adding more components. Stability of extracted components also helps to decide how many components should be studied. Specifically designed for neuroimaging data, ICASSO (somewhat confusingly, not an acronym), software investigates the reliability of ICA estimates by clustering and visualization, to visually and quantitatively select components with tight clusters, indicating good stability under different conditions (Himberg et al., 2004), to help improve the reliability of the estimated independent components.

For SNP data, an analogous method has been used (Chen et al., 2012), which runs ICA deposition with a large range of component numbers, and derives a peak indicating the most reliable results. In addition, *N*-fold cross-evaluation or sub-sampling can select the number of components with highly verified (repeated) results (Chen et al., 2012; i.e., components and correlations derived from p-ICA runs on subsamples are consistent with the best estimation of component number). Overall, there is no ground truth, although different estimation methods from those above can be combined and contrasted to make the final call, aiming at reliably and maximally extracting the information embedded in the data.

### Strategies for Dealing with Ethnicity

Large sample-size is desired for genetic and imaging studies, and researchers are loath to remove samples from analyses. However, another challenge derives from using heterogeneous, multi-racial/ethnic sample pools. Self-reported race/ethnicity is collected for genetic studies and confirmed/updated from genomic data or ancestry-informative markers. Through principal component analyses (PCA; Pearson, 1901; Price et al., 2006) or multidimensional-scaling (MDS), factors representing population structure can be identified and referenced to known population background from HapMap3 (Duan et al., 2008) or the 1000-genome project (Kuehn, 2008) using software applications, e.g., EIGENSTRAT and PLINK MDS function (Price et al., 2006; Purcell et al., 2007). These factors are continuous variables, not categorical races, reflecting genetic admixture in samples (Ma and Amos, 2012). Population structure should be controlled for in association studies of heterogeneous samples. One approach is to reconstruct genetic data after removing principal components associated with race (Price et al., 2006; Liu et al., 2010). p-ICA is applied to reconstructed genetic data and imaging phenotypes. Another approach is to covary out race factors when correlating component loadings derived from p-ICA (Chen et al., 2012). Finally, one should conduct separate analyses for homogenous subgroups (a single-race cohort), if sample size allows, as this serves as verification for heterogeneous large samples.

### Correcting Statistically for the Fact that Larger-Sized Genes Possess more SNPs

Unbalanced gene size has raised concerns in multivariate genetic studies based on gene-set or pathway enrichment tests (Mirina et al., 2012). Large genes generally possess more SNPs, even after SNP pruning in LD. When a test is based on the frequency of genetic variants of interest (i.e., count the total number of SNPs in a gene to be associated with a phenotype) against a null hypothesis, gene size may bias test significance, requiring adjustment. However, this affects factor-based analyses like p-ICA differently. Components/factors are extracted based on variances carried, and usually SNPs in LD (sometimes in a gene, but sharing a similar variation pattern), are grouped into one component. Association tests between genes and phenotypes are based on components, thus significance level is not inherently biased toward large genes. However, a large gene, possessing more SNPs, may carry a large variance across the sample, leading to a higher probability of it being extracted as a block, potentially missing smaller variance associations related to the phenotype. This is the same limitation facing p-ICA when dealing with very large-dimensional SNP data in a relatively small sample size (e.g., >100 K loci with 200 samples; Liu et al., 2008). Solutions (in addition to dimensionality reduction discussed above), include integrating prior knowledge as a reference, to guide ICA to search for components close to a provided reference function. This method, “p-ICA with reference,” was applied to a schizophrenia study (Chen et al., 2013a). p-ICA with reference is a hybrid data and hypothesis-driven approach; the selection of reference may derive from a gene of known relevant function or GWAS results.

### Understanding the Output of Parallel ICA, Including Significance Values

Parallel ICA extracts components for each data modality and finds the correlated pairs of components between the two modalities. *P*-values can be computed using standard general linear model approaches or bootstrapping for testing the phenotypes against the subject loading parameters output from p-ICA. The significance of correlation value should be corrected for all possible combination of pairs between the two modalities, e.g., if 5 components are extracted for phenotypic data, and 10 components for genetic data, then the significance should be corrected for 5 × 10 tests. Permutation test also can verify the significance of observed correlation in the data. Through permuting the sample, breaking down the coordinate between genetic and phenotypic data, random associations between genotype and phenotypes will produce a null distribution, based on which an empirical significance can be obtained for the observed correlation (Chen et al., 2012; Liu et al., 2012). The brain imaging and genetic patterns are weighted, thus even for identified linked patterns, certain highly weighted SNPs or brain regions may contribute more or less. They can also have negative or positive values, thus a brain region may be negatively associated with (or subtracting from) the overall pattern. Likewise, a given SNP may make a positive or negative contribution to the overall pattern. The sign in the case of the SNP data, taking into account how the SNP was originally coded (e.g., if AA, aa and aa are coded as -1, 0, and 1, then a positive weight indicates that allele A contributes negatively to the overall pattern), explains whether minor (A) or major (a) allele positively or negatively relates to the overall pattern, in other words leads to an increases or decrease of the pattern. Thus, once the set of weights is identified, it is straightforward to query any individual SNP for its positive or negative contribution to the overall pattern, which is helpful for the interpretation of any significant effects. Likewise, the subject loading parameters indicate the degree to which an individual subject contributes to the overall pattern of weighted brain regions or SNPs. This enables us to directly test for, e.g., group differences in the loading parameters using standard statistical tools (e.g., regression, ANOVA). A key difference between the massive univariate approach and p-ICA, is the testing is done at the network level, and thus significance values are corrected for the number of networks rather than the number of SNPs or voxels. Regarding component ordering, in p-ICA components are typically ordered by their inter-modal correlation. Other choices are possible as well such as their correlation with a variable of interest (e.g., diagnosis, symptoms, age). Sorting by eigenvalue is not particularly useful for ICA as ICA does not maximize the eigenvalues in an ordered way like in PCA.

### Is ICA Modeling Linkage Disequilibrium?-Limiting LD Structure and Detecting Epistasis

Linkage disequilibrium patterns describe population genetic structures, a haplotype distribution, primarily modeled as a pair-wise association for nearby SNPs. p-ICA is ultimately interested in SNP patterns correlating with phenotypes, e.g., brain data. These are driven both by correlation between SNPs (correlated SNPs are grouped into one pattern) and by allele distribution or portions thereof. Gene-gene interaction, epistasis, and unclear inter-genic regulatory mechanisms all can contribute to such patterns. LD structure can be limited by selecting one SNP per LD block, ICA can also extract SNP patterns related to phenotypic variation or disease. Regarding epistatic (non-linear) effects, though p-ICA is a linear mixing model, it is also a data-driven approach, thus it can still pick up the impact of epistatic effects within the estimated component patterns. For example, there is no requirement that loading parameters vary linearly among subjects, thus if a set of SNPs exhibit synergistic epistatic effects we should see a superlinear relationship of the loading parameters. Future work can more explicitly (and presumably more optimally) attempt to capture non-linear relationships, for example a non-linear ICA algorithm can be incorporated within p-ICA (Castro et al., 2015) or we could code the SNPs differently (for example two variables can be entered for each SNPs (coding for dominant/recessive models) to enable p-ICA to pick up dominant/recessive epistasis).

### Interpreting Rank Order of SNPs within a Component

Selecting top-weighted SNPs for interpretation of the genetic component involves: (1) z transformation of weights, and (2) thresholding top weighted markers. This threshold can reflect the most significant, for instance, 1%, | Z| > 3, or an inflection point of weight distribution (which decision depends on context as to whether this is sensible for a particular study). The thresholding decision is critical, especially for further pathway enrichment, because the lower the z-threshold defined, the greater the number of genes carried forward for pathway enrichment, raising the possibility of finding false positives. One exploratory strategy is to analyze pathways at different thresholds and report/emphasize those commonly significant. Various software suites use slightly different criteria for annotating genes.

Genetic annotation analyses can be used to aid interpretation of genetic findings. Testing for over-represented canonical pathways provides evidence about their known molecular function. Grouping top markers into clusters based on their known direct or indirect interactions helps identify associated genetic networks. Such tools include commercial software suites, e.g., Ingenuity Pathways Analysis (IPA)^6^, Pathway Studio^7^, and Metacore (formerly GeneGo ^8^), and freely available programs, e.g., DAVID^9^, ConsensusPathDB-human^10^, and PANTHER^11^. The widely used IPA is built on its own knowledge base, a repository of expertly curated biological interactions and functional annotations created from millions of individually modeled relationships between proteins, genes, complexes, cells, tissues, drugs, and diseases. It presents enrichment tests from different aspects and biological levels, including molecular/cellular function, system development, canonical pathway, network, and diseases/disorders. DAVID is freely available and widely used; providing flexible tests, including enrichment and classification, using combined databases, e.g., Kyoto Encyclopedia of Genes and Genomes (KEGG), GOterm, BioCarta (Huang da et al., 2009).

In brief, functional annotation is a means of identifying functional over-representation of genes associated with particular biological classifications to identify underlying biological themes. Software suites allowing such classifications have to account for the fact that genes/gene products often contribute to multiple biological pathways/systems and that hundreds or thousands of genes can act in parallel in a particular process. Gene ontologies are structured, controlled vocabularies that describe biological processes, molecular functions and cellular components associated with gene(s), i.e., the roles of genes. Such functional annotation/gene ontology pathways are relatively early “works in progress,” particular genes may participate in multiple biologic pathways, many remaining to be fully elucidated. Pathway analyses rely heavily on collected knowledge bases which have more or less complete up-to-date information on gene annotation, gene function, pathways, protein interaction, diseases association etc. Yet, knowledge we have is incomplete and rapidly changing (Khatri et al., 2012). Different software versions may produce different results, depending on available information (Henderson-Maclennan et al., 2010); reporting software versions used and analyzing data with two or more releases of the same software are strongly recommended.

Data-driven multivariate methods such as p-ICA have been usefully employed to assess mutual information between MRI and genetic data (Nymberg et al., 2013). Examples include (Liu et al., 2009a; Decoster et al., 2012; Meda et al., 2012, 2014). For example, in the Alzheimer’s disease Neuroimaging Initiative (ADNI) data set, structural imaging and genetic data from late-onset Alzheimer’s disease (LOAD) and healthy control subjects identified SNP components whose pathway analysis included genes already known to contribute to LOAD risk (e.g., APOE4) or involved in LOAD-related pathologic processes, including inflammation, type-2 diabetes, obesity and cardiovascular disease, plus significant novel genes. Analogously, p-ICA investigations of P300 amplitude identified SNPs from noradrenergic and dopaminergic genes in accord with prior models (Liu et al., 2009a), as well as SNP’s subsequently replicated by others (Decoster et al., 2012). Previous studies report that 7–25% of variance may be captured by a given SNP–fMRI pair, though this number is data-dependent.

## What Constitutes Replication in p-ICA?

This issue is important because of frequent non-replications in univariate genetic studies. Showing reproducibility is important especially when dealing with datasets containing multiple variables, also with independent data and in different labs. Approaches include formal cross-validation (e.g., leave-N-out) or split-half analysis. Characterizing robustness of findings to different software settings, data and preprocessing steps is important, and is one motivation for providing p-ICA tools via a freely downloadable software package, FIT^12^. One straightforward replication metric is examining results from either leave-N-out or split-sample replications, to assess derived gene components for either rank correlations or percent overlap from the most complex to the simplest level, i.e., molecular pathways, biological categories, genes and SNPs, respectively. Note, the components are data driven, so will not be identical, but quite often there are similar components, which are identified as mentioned above. If one has a need to use a component or components from one data set as a reference, that is a more formal constraint that the components should match, then one can estimate the same component from a new data set using a constrained ICA approach such as p-ICA with reference, except using the entire component as a reference instead of a smaller number of SNPs (Chen et al., 2013a).

## Outstanding Questions and Future Directions

Though we have attempted to address many typical questions that arise when employing a multivariate imaging genetics approach, there are some ongoing issues and emerging issues that we summarize briefly below:

Future research needs to confirm how best to utilize hybrid approaches (neither purely model-based nor data-driven), like p-ICA with reference (Chen et al., 2013a,b) and to incorporate >2 modalities (e.g., sMRI, fMRI, genetics; Vergara et al., 2014).

Another important area in need of clarification, is to address what can be gained by incorporating statistical properties such as sparsity (Cao et al., 2014; Lin et al., 2014a) or by linking genetics to newer fMRI approaches estimating dynamic changing connectivity patterns or states (Hutchison et al., 2013; Allen et al., 2014; Damaraju et al., 2014; Ma et al., 2014) that more cleanly characterize individuals or the impact of diseases (Rashid et al., 2013). In addition, current p-ICA software versions have not yet explicitly incorporated sparse regularization (Kohannim et al., 2012), but this is one direction for future development.

An area of considerable interest is whether stem cell models can incorporate p-ICA-derived pathways, and more broadly, how genome biology can move more effectively toward systems biology. Additionally, p-ICA can straightforwardly accommodate omics data sets of various types, although publications exploiting this approach in those venues lie in the future.

Attention needs to be directed as to how best to utilize new databases to illuminate in what sequences genes are expressed in development and spatially localized genomic information (e.g., the Allen human brain atlas^13^, or the NIH postmortem gene expression database/BrainSpan^14^).

Finally, in clarifying underlying genetic risk for neuropsychiatric disorders, researchers need to consider how can we best move toward a Research Domain Criteria (RDoC)-type model (Cuthbert and Insel, 2010), informed by biologic data across conventional psychiatric disorders, rather than models based on the American Psychiatric Association’s Diagnostic and Statistical Manual of Mental Disorders (American Psychiatric and Force, 2013; DSM) predicated on descriptive syndromes. The DSM comprises descriptions of diagnostic categories of major mental illnesses and their associated diagnostic criteria, classified primarily on symptoms and their associated clinical outcomes (e.g., functional impairment). This type of approach is reliable, but increasingly criticized for not being based on a valid biological foundation. In contrast, RDoC is a National Institute of Mental Health (NIMH)-based initiative for development of new means to classify psychopathology for research purposes, based on dimensions of observable behavior (e.g., working memory, fear circuitry) and their neurobiological underpinnings across multiple units of analysis (e.g., brain circuits, genes, behaviors). It is construed as cutting across psychiatric disorders conceived as traditional, symptom-defined syndromes (e.g., as operationalized in DSM), which are likely highly heterogeneous, and moving more toward a bottom-up redefinition based on underlying biology

## Concluding Summary

Despite general agreement that many complex medical and psychiatric diseases and complex quantitative traits are underpinned by multiple genes of individually small effect, the preponderance of genetic analyses are driven by univariate strategies that fail to capture a significant percentage of the relevant genetic variants or their interactions. Such univariate approaches have led to major advances, but are limited by the need for very large sample sizes and have limitations in their ability to illuminate the underlying molecular biological pathways needed to understand etiopathology, and hence to suggest novel treatments for disorders. Multi-variate approaches such as p-ICA offer promise in addressing some the above problems, but are a “work in progress” with some practical details still being fine-tuned and where true replication ability remains to be demonstrated. Particular advantages of multi-variate approaches include statistical efficiency, (dealing well with samples in the hundreds to low thousands), and an ability to deal with numerous and complex phenotypes in a flexible manner. Existing models range from fully data-driven approaches to “informed” hybrid models able, for example, to leverage results from GWAS. Multivariate approaches are especially at useful in identifying relationships among large, complex data sets while simultaneously performing data reduction procedures.

## Conflict of Interest Statement

The authors declare that the research was conducted in the absence of any commercial or financial relationships that could be construed as a potential conflict of interest.

## Footnotes

1 www.biocarta.com

2 www.genome.jp/kegg

3 www.med.unc.edu/pgc

4 www.med.unc.edu/pgc/psychchip

5 www.med.unc.edu/pgc

6 www.ingenuity.com

7 www.ariadnegenomics.com/products/pathway-studio

8 http://thomsonreuters.com/en/products-services/pharma-life-sciences/systems-biology.html

9 david.abcc.ncifcrf.gov

10 cpdb.molgen.mpg.de

11 www.pantherdb.org/

12 http://mialab.mrn.org/software/fit

13 http://human.brain-map.org/

14 www.brainspan.org
